# High genetic diversity of *Enterocytozoon bieneusi* in minks and raccoon dogs in northern China[Fn FN1]

**DOI:** 10.1051/parasite/2024071

**Published:** 2024-11-19

**Authors:** Nian-Yu Xue, Zhong-Yuan Li, Hai-Tao Wang, Ya Qin, Xue-Min Li, Qing-Yu Hou, Jing Jiang, Xing Yang, Hong-Bo Ni

**Affiliations:** 1 Department of Medical Microbiology and Immunology, School of Basic Medicine, Dali University Dali Yunnan Province 671099 PR China; 2 College of Life Sciences, Changchun Sci-Tech University Shuangyang Jilin Province 130600 PR China; 3 College of Veterinary Medicine, Yangzhou University Yangzhou Jiangsu Province 225000 PR China; 4 Guangxi Key Laboratory of Brain and Cognitive Neuroscience, College of Basic Medicine, Guilin Medical University Guilin the Guangxi Zhuang Autonomous Region 541199 PR China; 5 College of Veterinary Medicine, Qingdao Agricultural University Qingdao Shandong Province 266109 PR China; 6 College of Animal Science and Technology, Jilin Agricultural University Changchun Jilin Province 130118 PR China

**Keywords:** *Enterocytozoon bieneusi*, Prevalence, Genotypes, Minks, Raccoon dogs

## Abstract

*Enterocytozoon bieneusi*, a zoonotic pathogen prevalent in both humans and animals, is the most frequently diagnosed microsporidian species in humans and presents significant public health risks. However, data on the prevalence and genotypes of *E. bieneusi* in farmed minks (*Neovison vison*) and raccoon dogs (*Nyctereutes procyonoides*) in China are limited. Therefore, 275 minks (89 from Hebei Province, 57 from Heilongjiang Province, 109 from Liaoning Province, 20 from Shandong Province) and 235 raccoon dogs (114 from Hebei Province, 27 from Heilongjiang Province, 61 from Liaoning Province, 33 from Jilin Province) were examined for the prevalence and genotypes of *E. bieneusi* through sequence analysis of the internal transcribed spacer (ITS) region of the rRNA gene. The overall prevalence of *E. bieneusi* was 18.6% (95/510), with 10.5% (29/275) in farmed minks and 28.1% (66/235) in raccoon dogs. Ten genotypes (CHN-F1, genotype D, Type IV, EbpC, NCF2, NCF5, NCF6, Peru8, Henan V, and MJ5) were identified in minks and raccoon dogs. This study is the first to detect the CHN-F1, NCF2, NCF6, Peru8, and Henan V genotypes in minks and the NCF5, NCF6, and MJ5 genotypes in raccoon dogs. Additionally, the D, Type IV, and Peru8 genotypes, previously identified in humans, were also found in minks and raccoon dogs, suggesting that these animals could be potential sources of human microsporidiosis. These findings expand the understanding of *E. bieneusi*’s host distribution in China and contribute to the prevention of zoonotic *E. bieneusi* infections among farmed animals.

## Abstract (fr)

*Enterocytozoon bieneusi*, pathogène zoonotique répandu chez les humains et les animaux, est l’espèce de microsporidie la plus fréquemment diagnostiquée chez les humains et présente des risques importants pour la santé publique. Cependant, les données sur la prévalence et les génotypes d’*E. bieneusi* chez les visons (*Neovison vison*) et les chiens viverrins (*Nyctereutes procyonoides*) en élevage en Chine sont limitées. Par conséquent, 275 visons (89 de la province du Hebei, 57 de la province du Heilongjiang, 109 de la province du Liaoning, 20 de la province du Shandong) et 235 chiens viverrins (114 de la province du Hebei, 27 de la province du Heilongjiang, 61 de la province du Liaoning, 33 de la province du Jilin) ont été examinés pour la prévalence et les génotypes d’*E. bieneusi* par analyse de séquence de la région de l’espaceur interne transcrit (ITS) du gène de l’ARNr. La prévalence globale d’*E. bieneusi* était de 18,6 % (95/510), avec 10,5 % (29/275) chez les visons d’élevage et 28,1 % (66/235) chez les chiens viverrins. Dix génotypes (CHN-F1, génotype D, Type IV, EbpC, NCF2, NCF5, NCF6, Peru8, Henan V et MJ5) ont été identifiés chez les visons et les chiens viverrins. Cette étude est la première à détecter les génotypes CHN-F1, NCF2, NCF6, Peru8 et Henan V chez les visons et les génotypes NCF5, NCF6 et MJ5 chez les chiens viverrins. De plus, les génotypes D, Type IV et Peru8, précédemment identifiés chez l’homme, ont également été trouvés chez les visons et les chiens viverrins, suggérant que ces animaux pourraient être des sources potentielles de microsporidiose humaine. Ces résultats élargissent la compréhension de la distribution des hôtes d’*E. bieneusi* en Chine et contribuent à la prévention des infections zoonotiques à *E. bieneusi* chez les animaux d’élevage.

## Introduction

Microsporidia are highly diverse and specialized intracellular parasites that infect a wide range of hosts, including humans, domestic animals, and wildlife [14, 21]. The phylum Microsporidia includes around 45 families, 218 genera, and 1,700 species, of which 17 are known to infect humans. Among these, *Enterocytozoon bieneusi* accounts for over 90% of human microsporidial infections, often manifesting with symptoms such as diarrhea and lethargy [5, 12, 16, 18]. The transmission of *E. bieneusi* predominantly occurs via the fecal–oral pathway, typically through consuming contaminated food or water. Moreover, infection can result from direct exposure to infected persons or animals [12, 22].

As of now, approximately 820 distinct genotypes of *E. bieneusi* have been characterized through the amplification and sequencing of nucleotides in the internal transcribed spacer (ITS) region [11]. These genotypes are categorized into 15 phylogenetic groups, each with varying degrees of host specificity and zoonotic potential [8, 30]. Groups 1 and 2 are the primary phylogenetic taxa of zoonotic importance. Within these groups, genotypes D, EbpC, and IV in Group 1 are frequently implicated in both human and non-human infections as well as environmental contamination, while the dominant genotypes BEB4, BEB6, I, and J in Group 2 are commonly found in ruminants, non-ruminants, and humans [8, 14]. Conversely, most *E. bieneusi* genotypes within Groups 3–11 have a more restricted host range, posing a lower or uncertain risk to public health [14]. In 2018, Zhang was the first to report the infection rate and genotype distribution of *E. bieneusi* in minks. The study revealed that genotypes D, Peru11, and EbpC, which had previously been identified in humans, were also present in minks, raising concerns about the potential transmission of these genotypes to humans [27]. Additionally, many genotypes have been found in raccoon dogs, including CHN-R1, D, Type IV, Peru8, NCF2, NCR2, NCR1, and EbpA, all of which belong to phylogenetic Group 1 [17, 23, 28]. These results indicate that *E. bieneusi* present in minks and raccoon dogs could serve as a potential source of infection in humans.

As one of the world’s leading fur animal breeding countries, China had approximately 70.38 million fur animals in breeding as of 2015, including 32.4 million minks and 20.9 million raccoon dogs [28]. The main production areas are concentrated in the provinces of Shandong, Liaoning, Hebei, Heilongjiang, and Jilin, which together account for about 95% of the national breeding total. Nevertheless, information regarding the occurrence and genetic diversity of *E. bieneusi* in these two species in China is still sparse. The objective of this research was to investigate the prevalence and genotypic diversity of *E. bieneusi* in farm-raised minks (*Neovison vison*) and raccoon dogs (*Nyctereutes procyonoides*) in northern China, as well as to evaluate the possible risk of zoonotic transmission.

## Materials and methods

### Ethical standards

This study was approved by the Ethics Committee of Qingdao Agricultural University.

### Sample collection

This study was approved by the Qingdao Agricultural University. From October 2023 to June 2024, a total of 275 mink fecal samples were collected from the provinces of Hebei, Heilongjiang, Liaoning, and Shandong, and 235 raccoon dog fecal samples were collected from Hebei, Heilongjiang, Jilin, and Shandong (Table 1). The sampled farms were classified as small (≤ 1,000 animals), medium (> 1,000 and < 5,000 animals), and large (≥ 5,000 animals), based on their scale. Each fresh sample was collected immediately after defecation, placed in a single-use plastic bag, labeled with relevant information such as location, date, and species, stored in a cooler, and then transported to the laboratory. All fecal samples were stored at −20 °C until DNA extraction.

**Table 1:** Factors associated with the prevalence of *Enterocytozoon bieneusi* in farmed minks in northern China.

Factor	Category	No. positive/sample size	% (95% CI)	OR (95% CI)	*p*-value	Genotype (No.)
Region	Heilongjiang province	3/57	5.3 (0.7–11.2)	Reference	*p* < 0.001	CHN-F1 (2), D (1)
	Hebei province	19/89	21.4 (12.7–30.0)	4.9 (1.4–17.4)		CHN-F1 (5), IV (9), EbpC (1), NCF2 (1), Peru8 (2), Henan V (1)
	Liaoning province	7/109	6.4 (1.7–11.1)	1,2 (0.3–5.0)		CHN-F1 (4), IV (1), NCF2 (1), NCF6 (1)
	Shandong province	0/20	0 (–)	–		–
Collection year	2023	1/20	5% (−5.5 – 15.5)	Reference	*p* > 0.05	CHN-F1 (1)
	2024	28/255	11.0 (7.1–14.8)	2.3 (0.3–18.2)		CHN-F1 (10), D (1), IV (10), EbpC (1), NCF2 (2), Peru8 (2), Henan V (1), NCF6 (1)
Farm scale	Small	18/58	31.0 (18.8–43.3)	12.0 (3.8–37.7)	*p* < 0.001	CHN-F1(4), IV(9), EbpC (1), NCF2 (1), Peru8 (2), Henan V (1)
	Medium	4/111	3.6 (0.1–7.1)	Reference		CHN-F1 (1), D (1), NCF2 (1), NCF6 (1)
	Large	7/106	6.6 (1.8–11.4)	1.9 (0.5–6.7)		CHN-F1 (6), IV (1)
Age	Juvenile	1/30	3.3 (−3.5 – 10.2)	Reference	*p* > 0.05	CHN-F1 (1)
	Adult	28/245	11.4 (7.4–15.4)	3.7 (0.5–28.5)		CHN-F1 (10), D (1), IV (10), EbpC (1), NCF2 (2), Peru8 (2), Henan V (1), NCF6 (1)
Diarrhea	Yes	0/7	0 (–)	Reference	*p* > 0.05	–
	No	29/268	10.8 (7.1–14.6)	1.8 (0.1–33.2)		CHN-F1 (11), D (1), IV (10), EbpC (1), NCF2 (2), Peru8 (2), Henan V (1), NCF6 (1)
Total		29/275	10.5 (6.9–14.2)			CHN-F1 (11), D (1), IV (10), EbpC (1), NCF2 (2), Peru8 (2), Henan V (1), NCF6 (1)

CI, confidence interval; OR, odds ratio.

### DNA extraction and PCR amplification

In accordance with the manufacturer’s instructions, DNA was isolated from each stool sample using a Stool DNA Kit (Omega Bio-Tek Inc., Norcross, GA, USA) and subsequently stored at −20 °C. The prevalence and genotypes of farmed minks and raccoon dogs were assessed through nested PCR amplification targeting the ITS region. In the primary PCR, a 390 bp product was amplified using the forward primer F1 (5′–GGTCATAGGGATGAAGAG–3′) and the reverse primer R1 (5′–TTCGAGTTCTTTCGCGCTC–3′). In the secondary PCR, the forward primer F2 (5′–GCTCTGAATATCTATGGCT–3′) and the reverse primer R2 (5′–ATCGCCGACGGATCCAAGTG–3′) were used. Both groups underwent identical cycle conditions: initial denaturation at 94 °C for 5 min, followed by 35 cycles of 94 °C for 45 s, 55 °C for 45 s, and 72 °C for 1 min, and concluding with a final extension at 72 °C for 10 min. EX Taq enzyme (Takara, Shiga, Japan) was employed in all PCR reactions. Secondary PCR products were detected using 1.5% agarose gel electrophoresis, with GoldView™ (Solarbio, Beijing, China) staining.

### Sequence and phylogenetic analysis

The positive secondary PCR products were sent to Tongyong Biotech Company in Anhui, China, for bidirectional sequencing. The sequences obtained were compared with reference sequences in NCBI using BLAST (http://www.ncbi.nlm.nih.gov/BLAST/) and ClustalX 1.83 to identify *E. bieneusi* genotypes. A phylogenetic tree was generated using MEGA 6.0 (http://www.megasoftware.net/) through the Neighbor-Joining (NJ) method, applying the Kimura 2-parameter model, and supported by a bootstrap analysis with 1,000 iterations.

### Statistical analysis

The chi-square test in SPSS software (IBM Corp., Armonk, NY, USA) was employed to compare prevalence rates by region, species, year of collection, farm scale, and diarrhea status, with statistical significance set at *p* < 0.05. Additionally, odds ratios (ORs) and 95% confidence intervals (CIs) were calculated.

### Nucleotide sequence accession numbers

The nucleotide sequences obtained in this study have been submitted to the GenBank database under accession numbers PQ165127–PQ165142.

## Results

### Prevalence of *Enterocytozoon bieneusi*

The study found that the overall infection rate of *E. bieneusi* in all specimens was 18.6% (95/510), with 10.5% (29/275) in minks and 28.1% (66/235) in raccoon dogs (Table 1). The infection rate of *E. bieneusi* in minks across different provinces ranged from 0.0% to 21.4%, with the highest rate observed in Hebei Province at 21.4% (19/89), while no infections were detected in minks from Shandong Province (*p* < 0.001) (Table 1). For raccoon dogs, the infection rate ranged from 19.3% to 44.4% across different provinces, with Heilongjiang Province showing the highest rate at 44.4% (12/27) and Hebei Province the lowest at 19.3% (22/114) (*p* < 0.05) (Table 2). The prevalence of infection in farmed minks in 2023 was 5.0% (1/20), with no significant difference from the 11.0% (28/255) observed in 2024 (*p* > 0.05). The infection rate of raccoon dogs in 2023 was 45.5% (5/11), with no statistically significant difference from the 27.2% (61/224) observed in 2024 (*p* > 0.05). In addition, the infection rate on small-scale mink farms was 31% (18/58), significantly higher than the 3.6% (4/111) on medium-sized farms, and 6.6% (7/106) on large-scale farms (*p* < 0.001) (Table 1). For raccoon dogs, the infection rate on large-scale farms was 46.7% (7/15), significantly higher than the 28.9% (41/142) on small-scale farms, and 17.9% (12/67) on medium-sized farms (*p* < 0.05). The infection rate of *E. bieneusi* in juvenile minks was 3.3% (1/30), with no significant difference compared to 11.4% (28/245) in adult minks (*p* > 0.05). The infection rate of *E. bieneusi* in juvenile raccoon dogs was 18.2% (4/22), with no significant difference compared to 27.7% (56/202) in adult raccoon dogs (*p* > 0.05). Minks with diarrhea symptoms (0/7) were not detected to be infected with *E. bieneusi*, while the infection rate of minks without diarrhea symptoms was 10.8% (29/268) (*p* > 0.05). The infection rate of *E. bieneusi* in raccoon dogs with diarrhea symptoms was 16.7% (2/12), not significantly different from 28.7% (64/223) in raccoon dogs without diarrhea symptoms (*p* > 0.05) (Table 2).

**Table 2:** Factors associated with the prevalence of *Enterocytozoon bieneusi* in farmed raccoon dogs in northern China.

Factor	Category	No. positive/sample size	% (95% CI)	OR (95% CI)	*p*-value	Genotype (No.)
Region	Hebei province	22/114	19.3 (11.9–26.7)	Reference	*p* < 0.05	CHN-F1 (4), D (7), IV (3), NCF2 (3), NCF5 (1), NCF6 (2), Peru8 (2)
	Heilongjiang province	12/27	44.4 (24.4–64.5)	3.4 (1.4–8.2)		CHN-F1 (3), IV (2), NCF2 (6), Peru8 (1)
	Jilin province	10/33	30.3 (13.8–46.9)	1.8 (0.8–4.4)		CHN-F1 (1), D (1), IV (4), NCF2 (3), MJ5 (1)
	Liaoning province	22/61	36.1 (23.7–48.5)	2.4 (1.2–4.7)		CHN-F1 (10), D (10), IV (1), Peru8 (1)
Collection year	2023	5/11	45.5 (10.4–80.5)	2.2 (0.7–7.6)	*p* > 0.05	CHN-F1 (5)
	2024	61/224	27.2 (21.4–33.1)	Reference		CHN-F1 (13), IV (10), NCF2 (12), Peru8 (4), D (18), NCF5 (1), NCF6 (2), MJ5 (1)
Farm scale	Small	41/142	28.9 (21.3–36.4)	1.9 (0.9–3.8)	*p* < 0.05	CHN-F1(9), D (12), IV(8), NCF2 (6), NCF5 (1), NCF6 (2), Peru8 (2), MJ5 (1)
	Medium	12/67	17.9 (8.5–27.3)	Reference		CHN-F1(1), D (6), NCF2 (3), Peru8 (2),
	Large	7/15	46.7 (18.1–75.3)	4.0 (1.2–13.2)		CHN-F1(2), IV (2), NCF2 (3)
	Unknown	6/11	54.5 (19.5–89.6)	–		CHN-F1(6)
Age	Juvenile	4/22	18.2 (7.0–35.7)	Reference	*p* > 0.05	D (3), IV (1)
	Adult	56/202	27.7 (21.5–33.9)	1.7 (0.6–5.3)		CHN-F1 (12), IV (9), NCF2 (12), Peru8 (4), D (15), NCF5 (1), NCF6 (2), MJ5 (1)
	Unknown	6/11	54.5 (19.5–89.6)	–		CHN-F1(6)
Diarrhea	Yes	2/12	16.7 (−8.1 – 41.4)	Reference	*p* > 0.05	D (2)
	No	64/223	28.7 (22.7–34.7)	2.0 (0.4–9.4)		CHN-F1 (18), IV (10), NCF2 (12), Peru8 (4), D (16), NCF5 (1), NCF6 (2), MJ5 (1)
Total		66/235	28.1 (22.3–33.9)			CHN-F1 (18), IV (10), NCF2 (12), Peru8 (4), D (18), NCF5 (1), NCF6 (2), MJ5 (1)

CI, confidence interval; OR, odds ratio.

### *Enterocytozoon bieneusi* genotypes

Based on sequence analysis of the ITS region, ten distinct genotypes were identified: CHN-F1, D, Type IV, EbpC, NCF2, NCF5, NCF6, Peru8, Henan V, and MJ5 (Tables 1 and 2). Among these, CHN-F1 (*n* = 11) was the predominant genotype in minks, while CHN-F1 (*n* = 18) and D (*n* = 18) were predominant in raccoon dogs. The ITS sequence analysis revealed that the D (PQ165129, PQ165130), NCF2 (PQ165136, PQ165137), genotype IV (PQ165139, PQ165140), Peru8 (PQ165131, PQ165132), CHN-F1 (PQ165127, PQ165128), and NCF6 (PQ165133, PQ165135) genotypes shared 100% homology with previously identified genotypes in Chinese pigs (MK778893), Australian Eastern grey kangaroos (MG976814), Chinese foxes (MN029060), Chinese raccoon dogs (MN747470), Chinese raccoon dogs and foxes (KU847359 and KR998501), and Chinese foxes (KT750159).

Additionally, Henan V (PQ165138) in mink samples exhibited 100% homology with sequences identified in Chinese cobras (KJ651439), and EbpC (PQ165141) also shared 100% homology with sequences identified in Chinese coypus (MT557704). Similarly, NCF5 (PQ165134) in raccoon dog samples showed 100% homology with sequences found in Chinese foxes (KT750158), while MJ5 (PQ165142) exhibited 100% homology with sequences identified in Chinese Asiatic black bears (MK547519).

### Phylogenetic relationship of *E. bieneusi*

**Figure 1:**
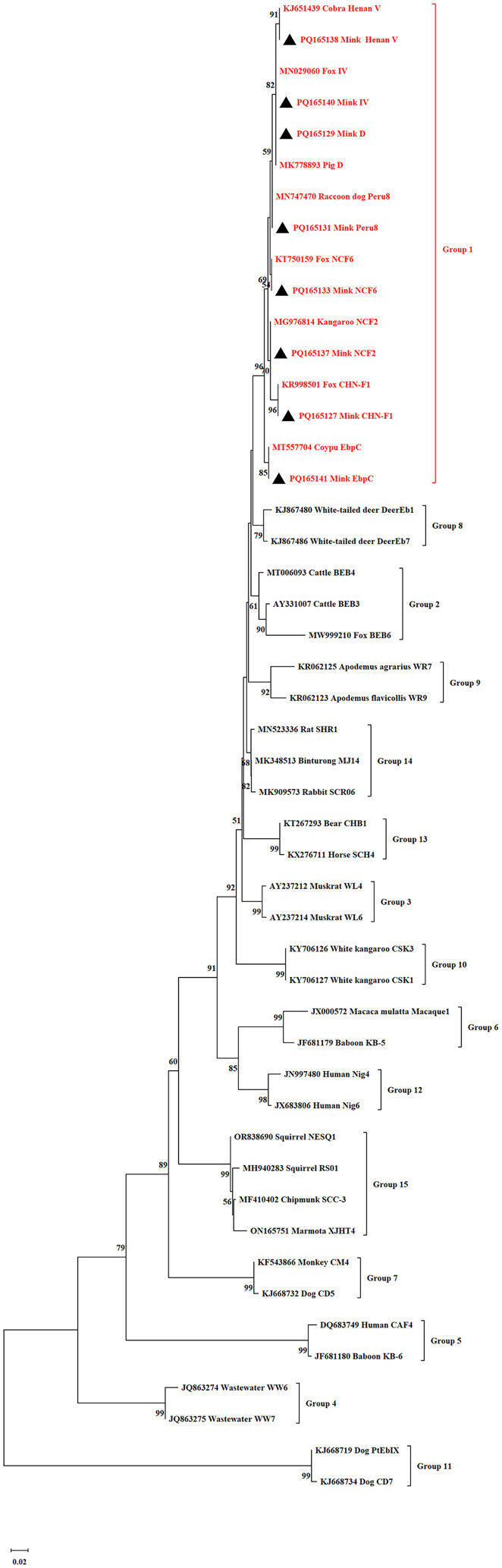
Phylogenetic relationships among *Enterocytozoon bieneusi* isolates from minks were determined using a neighbor-joining analysis based on ITS nucleotide sequences. Cluster reliability was assessed through bootstrap analysis with 1,000 replicates, displaying values above 50% beside the nodes. Black triangles mark the known ITS genotypes identified in this study.

**Figure 2:**
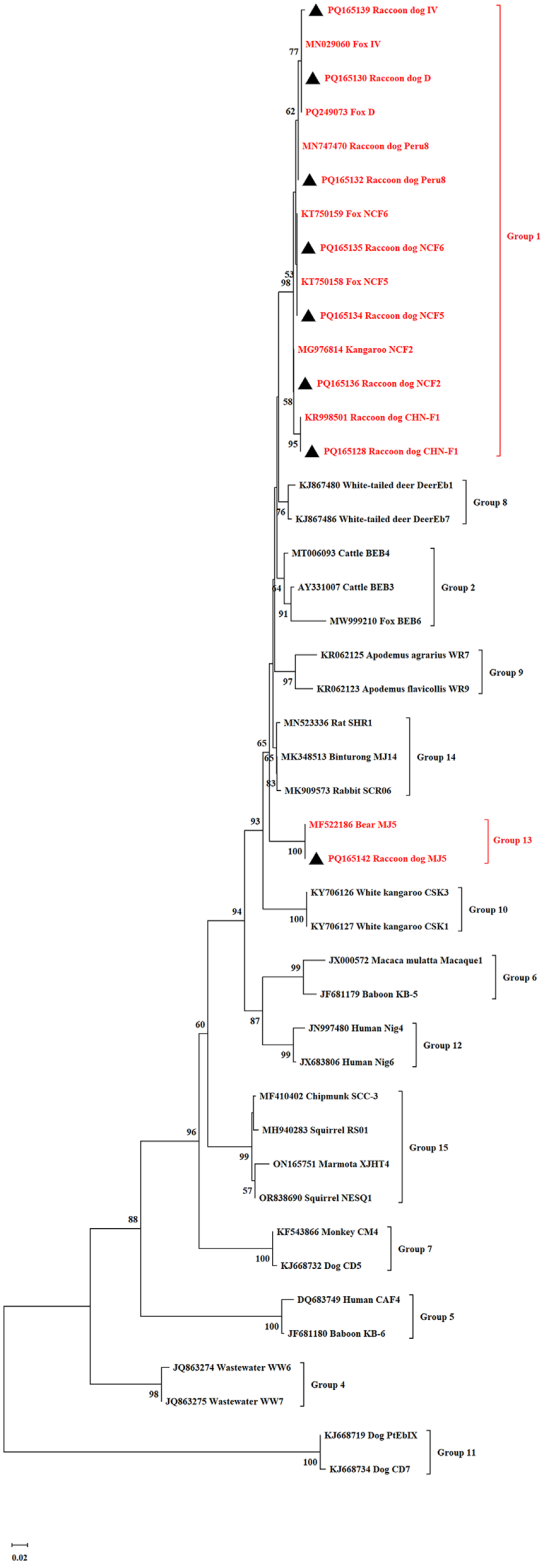
Phylogenetic relationships among *Enterocytozoon bieneusi* isolates from raccoon dogs were determined using a neighbor-joining analysis based on ITS nucleotide sequences. Cluster reliability was assessed through bootstrap analysis with 1,000 replicates, displaying values above 50% beside the nodes. Black triangles mark the known ITS genotypes identified in this study.

A phylogenetic tree based on the ITS nucleotide sequences of *E. bieneusi*, as shown in Figures 1 and 2, revealed that CHN-F1, D, Type IV, EbpC, NCF2, NCF5, NCF6, Peru8, and Henan V belong to Group 1, whereas MJ5 belongs to Group 13.

## Discussion

There is a relatively limited amount of research on the global epidemiology of *E. bieneusi* in minks and raccoon dogs. In this study, the overall infection rate of *E. bieneusi* in farmed minks from northern China was 10.5% (29/275), which is higher than the 5.6% (12/214) reported in farmed minks from Xinjiang [28] and the 4.1% (23/559) in farmed minks from Heilongjiang and Jilin provinces [4]. We found that minks in Hebei Province had the highest infection rate of *E. bieneusi* at 21.4% (19/89), compared to 6.4% (7/109) in Liaoning Province, 5.3% (3/57) in Heilongjiang Province, and 0% (0/20) in Shandong Province. Interestingly, a 2016 study on the epidemiology of *E. bieneusi* in northern China also found that minks in Hebei Province had the highest infection rate, while no infection was found in minks from Shandong Province [27]. This suggests that the prevalence of *E. bieneusi* in minks across different regions is closely related to geographical location and the hygiene conditions of breeding farms. In this study, the overall infection rate of *E. bieneusi* in raccoon dogs in northern China was 28.1% (66/235), which is lower than the 40.2% (35/86) reported in wild raccoon dogs in Poland [19] and the 35.4% (17/48) reported in wild raccoon dogs in South Korea [1]. Reported infection rates of *E. bieneusi* in farmed raccoon dogs in China range from 2.6% to 22.3% [17, 23, 24, 28, 29], while the infection rate found in this study was higher. Among the four provinces, Heilongjiang recorded the highest *E. bieneusi* infection rate in raccoon dogs at 44.4% (12/27), followed by Liaoning at 37.3% (22/59), Jilin at 30.3% (10/33), and Hebei at 19.3% (22/114). The prevalence of *E. bieneusi* in raccoon dogs is influenced by various factors, including geographical region, feeding conditions, and animal welfare. The infection rate of *E. bieneusi* in farmed minks on small-scale farms was 31% (18/58), significantly higher than the 3.6% (4/111) on medium-sized farms and 6.6% (7/106) on large-scale farms. This difference may be attributed to the higher animal density on small-scale farms, which facilitates the spread of *E. bieneusi*. The infection rate on large-scale farms for raccoon dogs was the highest, reaching 46.7% (7/15), compared to 28.9% (41/142) on small-scale farms and 17.9% (12/67) on medium-sized farms. The elevated infection rate on large-scale raccoon dog farms may be due to factors such as small sample size, poor feeding conditions, and water source contamination by *E. bieneusi*. In the future, we will increase the sampling size for large-scale farms and conduct further investigations into the water sources to confirm the infection status of *E. bieneusi*. In addition, the infection rates of *E. bieneusi* in adult minks and raccoon dogs were 11.4% (28/245) and 27.7% (56/202), respectively both higher than the infection rates in juvenile minks and raccoon dogs, which were 3.3% (1/30) and 18.2% (4/22). This finding is consistent with previous studies [3]. The infection rate of *E. bieneusi* was found to be lower in minks and raccoon dogs with diarrhea symptoms than in those without diarrhea symptoms. Although these asymptomatic animals did not exhibit diarrhea, they were still capable of continuously shedding infectious spores, complicating disease prevention efforts. Therefore, farms that raise minks and raccoon dogs should consider enhancing epidemiological monitoring of non-diarrheal animals for *E. bieneusi*.

In this study, we identified ten genotypes of *E. bieneusi* through sequencing: CHN-F1, D, EbpC, Type IV, NCF2, NCF5, NCF6, Peru8, Henan V, and MJ5, with CHN-F1 being the most prevalent. The CHN-F1, D, Type IV, EbpC, NCF2, NCF5, NCF6, Peru8, and Henan V genotypes all belong to Group 1, which carries potential zoonotic risk, whereas the MJ5 genotype is classified in Group 13. Previously, the D, Type IV, and EbpC genotypes had been detected in minks [26]. However, this study is the first to report the presence of the CHN-F1, NCF2, NCF6, Henan V, and Peru8 genotypes in minks. Additionally, while previous studies reported raccoon dogs infected with the CHN-F1, D, Type IV, NCF2, and Peru8 genotypes [17, 23], this study is the first to identify the NCF5, NCF6, and MJ5 genotypes in raccoon dogs. The CHN-F1 genotype was first identified in foxes in Heilongjiang and Jilin provinces, northern China [29], later detected in raccoons in southern China [23], and subsequently found in pigeons in central Europe [6]. This suggests that the CHN-F1 genotype has a broad host range and poses a potential risk for cross-species transmission. Genotype D has been identified in 91 host species across 40 countries, while genotype EbpC has been detected in 43 host species across 15 countries, indicating both genotypes have a broad host range and extensive geographical distribution [7, 28]. In China, these genotypes have been detected in diverse populations across 26 provinces and cities, as well as in a variety of wild, domestic, and companion animals [20]. Additionally, genotype D is the most common cause of human infections, followed by genotype EbpC [9]. Type IV has been frequently detected in a wide range of animal species, including nonhuman primates, dogs, cats, cattle, rabbits, various rodents, deer, foxes, birds, and snakes [13]. The NCF2, NCF5, and NCF6 genotypes were first reported in farmed foxes in northern China [26], with NCF2 later detected in raccoon dogs [23]. Although these genotypes belong to zoonotic Group 1, they have not yet been detected in humans. The Peru8 genotype is frequently detected in both humans and animals [12], whereas the Henan V genotype has been identified in captive snakes, dogs, and macaques [10, 15, 25]. The MJ5 genotype, previously identified in pet birds and black bears, was detected in raccoon dogs for the first time in this study, thereby expanding its known host range. The genotypes D, EbpC, Type IV, and Peru8 identified in minks and raccoon dogs in this study are also the most commonly detected genotypes in humans and are frequently reported in livestock, wild animals, and various water sources. This indicates that minks and raccoon dogs could be potential sources of *E. bieneusi* infection in humans, with both humans and animals potentially becoming infected through the consumption of contaminated water [2].

In conclusion, this study investigated the prevalence and genetic diversity of *E. bieneusi* in minks and raccoon dogs in northern China. The results indicated that the overall infection rate was 10.5% (29/275) in minks and 28.1% (66/235) in raccoon dogs. Ten genotypes were identified: CHN-F1, D, Type IV, EbpC, NCF2, NCF5, NCF6, Peru8, Henan V, and MJ5. This study is the first to detect the CHN-F1, NCF2, NCF6, Peru8, and Henan V genotypes in minks, and the NCF5, NCF6, and MJ5 genotypes in raccoon dogs. The detection of zoonotic *E. bieneusi* genotypes in minks and raccoon dog feces indicates that *E. bieneusi* spores may contaminate the environment, posing a potential public health risk. Furthermore, it is essential to implement effective measures to prevent outbreaks of waterborne microsporidiosis.
